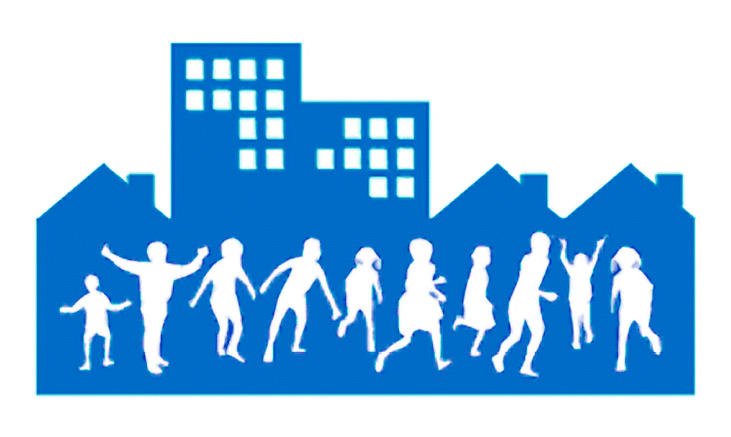# EHPnet: Community Environmental Health Resource Center

**Published:** 2005-05

**Authors:** Erin E. Dooley

The U.S. Environmental Protection Agency defines environmental justice as “the fair treatment of all people regardless of race, color, national origin, or income with respect to the development, implementation, and enforcement of environmental laws, regulations, and policies.” Among other goals, environmental justice activists work to better the living conditions of low-income communities, which often bear a disproportionate burden of environmental health hazards and the resulting health problems. One group working to improve housing for low-income communities around the nation is the Community Environmental Health Resource Center (CEHRC, pronounced “search”), based in Washington, DC. The CEHRC website at **http://www.cehrc.org/** gives communities the tools to document housing deficiencies as well as to pursue corrective and preventive action. A number of the pages on the site are available in Spanish.

One section of the website is devoted to exposing health hazards in housing. This section provides discussion papers and other documents that describe why it is important for community members to become involved in identifying the hazards in their homes. There is also guidance to help advocates work effectively and responsibly with community residents. This guidance offers insights into how to avoid adverse outcomes (such as faulty repairs that exacerbate hazards), protect residents’ rights and privacy, and other ethical considerations.

At the core of the site is the Tools for Detecting Hazards section, which concentrates on five main health threats: lead, carbon monoxide, cockroaches, mold/moisture, and radon. For each threat the site provides background materials, step-by-step sampling instructions and checklists, decision guides to help determine whether testing is warranted in certain situations, and other materials. The lead segment includes specific information for various routes of exposure: dust, paint, soil, and water. The Tools section also provides thorough instructions in both English and Spanish for conducting a visual survey of a residence and preparing a visual survey report.

The How Communities Create Solutions section has information on how to actually enact change within a community. The Data as a Catalyst for Change portion discusses how data can be used to back up advocacy campaigns. It also provides a discussion paper on strategies for holding property owners and government agencies responsible, as well as an overview of models of social change to help organizations define their missions and goals. The Tools for Change page provides time-tested methods to create change through recruiting and training volunteers, accessing political resources, and fundraising. Other portions include information on tenants’ rights, case studies, and the basics of developing and understanding policy and legislation.

## Figures and Tables

**Figure:**